# Supplementary material for: The evaluation of COVID-19 prediction precision with a Lyapunov-like exponent
Source: PLoS One. 2021 May 28;16(5):e0252394. doi: 10.1371/journal.pone.0252394 (PMC8162606; doi:10.1371/journal.pone.0252394)
Supplement: Attachment — Submitted filename: Response to reviewers.docx [file pone.0252394.s002.docx]

**Author’s response to reviewers**

**Please include a copy of Table 3 which you refer to in your text on pages 5 and 6.  (NB. there are currently two tables labelled as Table 2.)**

**It was corrected, and the last Table was labelled as Table 3.**

**Reviewer #1:**

**1. Literature survey of the paper is too weak, the authors are advised to revise accordingly. Authors are advised to revise the methodology section and suggesting to provide comparative analysis to connect regarding the predictions precision/**

**Introduction and literature survey were significantly expanded.**

**2. English and Grammatical mistakes needs to be improved throughout the paper.**

**The paper was proofread by a professional proofreader.**

**3. References needs to be in the format and authors are advised to refer the below articles and include the comparative analysis , build literature survey from the below papers**
**-> Gomathi, S., Kohli, R., Soni, M., Dhiman, G. and Nair, R. (2020), "Pattern analysis: predicting COVID-19 pandemic in India using AutoML", World Journal of Engineering, Vol. ahead-of-print No. ahead-of-print. https://doi.org/10.1108/WJE-09-2020-0450

-> K. Chandra, G. Kapoor, R. Kohli and A. Gupta, "Improving software quality using machine learning," 2016 International Conference on Innovation and Challenges in Cyber Security (ICICCS-INBUSH), Greater Noida, India, 2016, pp. 115-118, doi: 10.1109/ICICCS.2016.7542340.**

**The first paper mentioned above was included into the text (and a literature survey) and References. Second one is, in our opinion, too distant from the topic of the paper.**

**References were correctly formatted. Please note, that the track changes were made before the manuscript was transformed into PLoS ONE template.**

**All changes in the manuscript were highlighted in yellow in the pdf version.**

**Thank you for your comments and remarks.**

**Reviewer #2:**

**1. Abstract has to be re-written. The authors have to start with discussion on the importance of the topic, limitations of existing works and then introduce their solutions.**

**The abstract was rewritten accordingly.**

**2. What are the limitations of the existing works that motivated the researchers to carry out the current work?**

**Limitations of the proposed approach to predictions’ precision measurement were added into new Section 4 - Discussion.**

**3. List out the major contributions of the current work.**

**Two major advantages of the current work are now mentioned in Abstract, thorough the text of sections, and also they are summarized in Conclusions.**

**4. Some of the recent works on covid-19 and other disease predictions such as the following can be discussed in the paper "An Incentive Based Approach for COVID-19 using Blockchain Technology, Deep learning and medical image processing for coronavirus (COVID-19) pandemic: A survey, Early detection of diabetic retinopathy using PCA-firefly based deep learning model".**

**The second paper mentioned above was included into the text (and a literature survey) and References. First and third one are, in our opinion, too distant from the topic of the paper.**

**5. A detailed discussion on authors' inferences on the results obtained has to be discussed in the paper.**

**Results of a comparison of COVID-19 predictions is discussed in newly added Section 4: Discussion and limitations of the proposed approach.**

**6. Discuss about the limitations of the existing works.**

**Limitations were added into new Section 4 - Discussion.**

**All changes in the manuscript were highlighted in yellow in the pdf version. Please note, that the track changes were made before the manuscript was transformed into PLoS ONE template.**

**Thank you for your comments and remarks.**
